# Work-Related Musculoskeletal Disorders Among Medical Practitioners in the Hospitals of Al’Qassim Region, Saudi Arabia‏

**DOI:** 10.7759/cureus.8382

**Published:** 2020-05-31

**Authors:** Yasser Alwabli, Moath A Almatroudi, Meshari A Alharbi, Muath Y Alharbi, Sultan Alreshood, Faisal A Althwiny

**Affiliations:** 1 Orthopaedics, College of Medicine, Qassim University, Buraidah, SAU; 2 Orthopaedics, Unaizah College of Medicine, Qassim University, Unaizah, SAU

**Keywords:** musculoskeletal, wmsd, healthcare practitioners, risk factor

## Abstract

Introduction

Work-related musculoskeletal disorders (WMSDs) have a significant impact on the workplace; they have been growing as a problem in our population, especially among healthcare practitioners. The aim of the study was to determine the prevalence of the condition, evaluate the WMSDs experienced by practitioners in different specialties in different hospitals in the Al’Qassim region, and study various risk factors that contribute to the development of WMSDs.

Methods

After gaining the Institutional Review Board (IRB) approval, a cross-sectional study was conducted among all medical care practitioners at hospitals in the Al’Qassim region. Data were collected using a validated, standardized, and self-administered questionnaire. The data were analyzed using the SAS software version 9.4 (SAS Institute Inc., Cary, NC).

Results

The study result revealed that out of 382 participants, just over half (209, 54.7%) experienced WMSDs. Among them, 103 (27.54%) were males, and 271 (72.54%) were females. The average age of participants was 31.25 ±6.82 years. Participants with experience of 6-10 years were twice as likely to develop WMSDs compared to participants with fewer years of experience [odds ratio (OR): 2.342; 95% confidence interval (CI): 1.062-5.168; p = 0.0350]. There was no significant difference in terms of past medical history between the two groups except for the history of having low back pain, which was more common in participants with WMSDs (77.59% versus 22.41%; p: <0.0001), and neck pain (74.19% versus 25.81%; p = 0.0003). Almost all job risk factors varied significantly between the groups (p: <0.05). Prevalent risk factors included performing the same task over and over again (134, 37.96%), treating an excessive number of patients in one day (127, 35.88%), and working in the same positions (126, 35.8%).

Conclusions

Based on this study findings, we can conclude that WMSDs affect a high proportion of healthcare professionals; the female gender and more than six years of experience were found to be major predictors for WMSDs. Pain in the lower back, shoulders, and neck were the most frequently reported musculoskeletal complaints (MSCs). Hence, we recommend the incorporation of musculoskeletal disorder prevention programs in the hospitals' educational programs as they will reduce the rate of WMSDs and ensure the health and well-being of healthcare practitioners.

## Introduction

Musculoskeletal complaints (MSCs) in the workplace have a significant impact on the health of healthcare practitioners. They have been growing as a problem in the population, especially among healthcare workers, and they are an important cause of temporary work disability [1-3]. MSCs include a wide variety of degenerative and inflammatory disorders that can affect the joints, muscles, tendons, ligaments, supporting blood vessels or even peripheral nerves, and susceptible body parts such as lower back, neck, shoulders, arms, forearms, hands, and lower extremities [4]. The MSCs have a significant negative impact on healthcare practitioners during their duty. Physicians, nurses, physiotherapists, and other healthcare practitioners face repetitive trauma and constant strains in their routine patient care activities that cause chronic illnesses and musculoskeletal injuries [5]. Work-related musculoskeletal disorders (WMSDs) are defined as musculoskeletal disorders that result from an event that is related to work [6]. Working as a healthcare provider is identified as an important risk factor for WMSDs [3,7-9]. Healthcare workers have a higher prevalence of low back pain compared to other hospital and industrial workers [10]. A study about WMSDs that was done in Saudi Arabia among dental professionals showed that 85% experienced MSCs in some form [11]. Another study conducted on WMSDs among healthcare practitioners in India showed that 50.7% of the participants experienced MSCs [3]. Also, a study in China showed that 70% of nurses experienced MSCs [12]. Back, neck, knee, and shoulder complaints are most commonly seen among healthcare practitioners [3,13-15]. The aim of the study was to determine the prevalence of this condition, evaluate the WMSDs experienced by practitioners in different specialties in different hospitals in the Al’Qassim region, and study the different risk factors that contribute to the development of WMSDs.

## Materials and methods

After gaining the institutional Review Board (IRB) approval, a cross-sectional study was conducted among the medical practitioners in the Al’Qassim province in 2019. The study was carried out at the following three governmental hospitals in the Al’Qassim province: King Saud Hospital, Unaizah; Buraidah Central Hospital, Buraidah; and Ar Rass General Hospital, Ar Rass. The hospitals were chosen by a simple random technique. The sample size was calculated by using OpenEpi software version 3, and the result was as follows: n = [DEFF*Np(1-p)]/ [(d2/Z21-α/2*(N-1)+p*(1-p)] =375. All medical care practitioners at Al’Qassim hospitals, including physicians, nurses, lab workers, and other allied medical practitioners, were included in the study. All hospital administration staff were excluded.

Data were collected using a validated pretested questionnaire used in the study conducted by Yashwant S et al. 2014 [3]. The study tool validation was approved by experts and by the Alpha-Cronbach test. The questionnaire was in English and consisted of different standardized questionnaires. The first part of the questionnaire consisted of demographic data, lifestyle, and occupational history. The second part comprised the Self-reported Ergonomic Hazards at Workstation Questionnaire. The third part consisted of the Job Factors Questionnaire, which was administered using an ordinal scale of 0-10 to determine if the symptoms were work-related. Finally, the last part comprised the Nordic Musculoskeletal Questionnaire (NMQ) to identify the presence of self-reported musculoskeletal symptoms in the preceding 12 months (Table 6, Appendix).

A pilot study was done on 10% of our sample size (38 participants; 19 male and 19 female) before starting our actual study in order to test the validity of our questionnaire and to estimate the timing needed for each participant to complete the questionnaire. Specific data were collected as identified in the questionnaire papers and were listed on Excel spreadsheets (Microsoft, Redmond, WA). The data were analyzed using the SAS system version 9.4. (SAS Institute Inc., Cary, NC). Questionnaire scores were calculated according to the definitions described in the questionnaire. For categorical variables, frequencies and percentages were calculated, whereas, for continuous variables, results were presented as mean ±standard deviation (SD), or median ±interquartile range (IQR) in case of skewed data. Association between categorical variables was analyzed using the chi-squared test (χ2), whereas in cases of zero or small cells, Fisher’s exact test was implemented instead. A two-sample t-test was used for the normally distributed continuous variables; otherwise, a Mann-Whitney (Wilcoxon) two-sample test was implemented. Multivariable logistic regression analysis was performed. Odds ratios (ORs) with 95% confidence intervals (CIs) were expressed relative to a reference baseline category. A p-value of less than 0.05 was considered statistically significant. All results were summarized in tables and figures.

## Results

A total of 382 participants were included based on the inclusion and exclusion criteria; 103 (27.54%) were males, 271 (72.54%) were females; the gender of eight participants was not provided in their answers to the questionnaire. The average age of the participant was 31.25 ±6.82 years. Among the participants, 209 (54.7%) had experienced WMSDs. The age, gender, and other participant demographics are presented in Table 1.

**Table 1:** Comparison of demographic factors between participants with WMSDs and without WMSDs WMSDs: work-related musculoskeletal disorders; IQR: interquartile range ^*^If the numbers do not add up to the total, it represents missing data, and the shown % figure represents a valid percentage ^a^Mann-Whitney (Wilcoxon) two-sample test was implemented for these variables ^b^Fisher’s exact test was implemented for these variables

Demographics	^*^Total participants (n=382)	Participants with WMSDs (n=209)	Participants without WMSDs (n=173)	P-value	Frequency of missing data in demographic categories (n)	Frequency of missing data in participants with and without WMSDs (n)	Total frequency of missing data (n)
^a^Age, median (IQR)^a^	29.00 (8)	29.00 (8)	30.00 (9)	0.2806	-	-	-
^a^BMI, median (IQR)	24.00 (5.54)	24.32 (6)	24.57 (5.25)	0.6606	-	-	-
^a^Number of shifts, median (IQR)	8.00 (10)	3.00 (1)	3.00 (0)	0.6415	-	-	-
^a^Duration of 1 shift, median (IQR)	8.00 (5.5)	8.00 (1)	8.00 (0)	0.0135	-	-	-
Sex				0.2584	8		
Male, n (%)	103 (27.54%)	52 (50.98%)	50(49.02%)		-	1	9
Female, n (%)	271 (72.46%)	153 (57.52%)	113 (42.48%)		-	5	13
Marital status				0.0876	12		
Married, n (%)	209 (56.49%)	108 (52.17%)	99 (47.83%)		-	2	14
Unmarried, n (%)	161 (43.51%)	96 (61.15%)	61 (38.85%)		-	4	16
Current occupation					50		
Physician, n (%)	41 (12.42%)	23 (56.1%)	18 (43.9%)	0.8753		0	50
Nurse, n (%)	215 (64.95%)	123 (58.3%)	88 (41.7%)	0.6449	-	4	54
Physiotherapist, n (%)	19 (5.76%)	13 (68.42%)	6 (31.58%)	0.3096	-	0	50
^b^Radiologist, n (%)	9 (2.72%)	4 (44.44%)	5 (55.56%)	0.5038	-	0	50
Lab technician, n (%)	15 (4.55%)	7 (46.67%)	8 (53.33%)	0.3971	-	0	50
Pharmacist, n (%)	23 (6.97%)	15 (65.22%)	8 (34.78%)	0.4219	-	0	50
^b^Other occupations, n (%)	10 (3.03%)	3 (33.33%)	6 (66.67%)	0.1787	-	1	51

There was no significant difference between groups in terms of past medical history except for the history of having low back pain, which was more common in participants with WMSDs (77.59% versus 22.41%; p: <.0001), and neck pain (74.19% versus 25.81%; p = 0. 0003). In this study population, 30.7% of the participants were involved in physical activities such as sports and exercises routinely. The overall number of smokers was 26 (6.86%), of which 14 participants experienced WMSDs, and 12 did not.

Higher rates of almost all the self-reported ergonomic hazards at workstation were seen in participants with neck flexion of more than 20 degrees among WMSD participants (169, 64.50%) compared to those who did not have WMSDs (93, 35.5%) (p = 0.0001) (Table 2).

**Table 2:** Self-reported ergonomic hazards at workstation WMSDs: work-related musculoskeletal disorders ^*^If the numbers do not add up to the total, it represents missing data, and the shown % figure represents a valid percentage

Workstation hazards	^*^Total participants (n=382), n (%)	Participants with WMSDs (n=209), n (%)	Participants without WMSDs (n=173), n (%)	P-value	Frequency of missing data in workstation hazards categories (n)	Frequency of missing data in participants with and without WMSDs (n)	Total frequency of missing data (n)
Neck flexion of more than 20 degrees	263 (71.27%)	169 (64.50%)	93 (35.5%)	0.0001	13	4	17
Arm level higher than shoulder	190 (51.63%)	119 (63.64%)	68 (36.36%)	0.0053	14	4	18
Repetitive work of more than 4 minutes	270 (73.77%)	164 (61.42%)	103 (38.58%)	0.0020	16	4	20
Forceful work	210 (56.91%)	136 (65.70%)	71 (34.30%)	0.0001	13	4	17
Forward bending of the trunk	254 (69.02%)	162 (64.54%)	89 (35.46%)	0.0001	14	4	18
Lateral bending or twisting of the trunk	196 (53.41%)	127 (65.80%)	66 (34.20%)	0.0001	15	4	19
Prolonged sitting of more than 20 minutes	190 (51.63%)	123 (65.08%)	66 (34.92%)	0.0005	14	4	18
Prolonged standing of more than 20 minutes	292 (79.78%)	175 (60.76%)	113 (39.24%)	0.0017	16	4	20
Lifting, pulling or pushing	251 (68.02%)	146 (58.87%)	102 (41.13%)	0.1724	13	4	17

Job risk factors for different categories are shown in Table 3. Almost all job risk factors significantly varied among the different groups (p: <0.05). Performing the same task over and over again (134 participants, 37.96%), treating an excessive number of patients in one day (127 participants, 35.88%), and working in the same positions (126 participants, 35.8%) were the top three job risk factors recorded.

**Table 3:** Self-reported job risk factors among all participants WMSDs: work-related musculoskeletal disorders ^*^If the numbers do not add up to the total, it represents missing data, and the shown % figure represents a valid percentage

Job risk factor	^*^Total participants (n=382), n (%)	Participants with WMSDs (n=209), n (%)	Participants without WMSDs (n=173), n (%)	P-value	Frequency of missing data in job risk factor categories (n)	Frequency of missing data in participants with and without WMSDs (n)	Total frequency of missing data (n)
Performing the same task over and over again				<0.0001	29	4	33
No problem	50 (14.16%)	10 (20%)	40 (80%)				
Minimal to moderate	169 (47.88%)	95 (57.23%)	71 (42.77%)				
Major problem	134 (37.96%)	93 (69.92%)	40 (30.08%)				
Treating an excessive number of patients in 1 day				0.0001	28	4	32
No problem	58 (16.38%)	23 (39.66%)	35 (60.34%)				
Minimal to moderate	169 (47.74%)	87 (52.41%)	79 (47.59%)				
Major problem	127 (35.88%)	89 (70.63%)	37 (29.37%)				
Performing manual orthopedic techniques (joint mobilizations, soft tissue mobilization)				0.0238	42	4	46
No problem	137 (40.29%)	66 (48.53%)	70 (51.47%)				
Minimal to moderate	168 (49.41%)	106 (63.86%)	60 (36.14%)				
Major problem	35 (10.29%)	21 (61.76%)	13 (38.24%)				
Not enough rest breaks or pauses during the workday				0.0007	26	4	30
No problem	65 (18.26%)	24 (37.5%)	40 (62.5%)				
Minimal to moderate	190 (53.37%)	110 (58.2%)	79 (41.8%)				
Major problem	101 (28.37%)	67 (67.68%)	32 (32.32%)				
Working in awkward and cramped positions				<0.0001	32	4	36
No problem	65 (18.57%)	21 (32.81%)	43 (67.19%)				
Minimal to moderate	214 (61.14%)	126 (59.15%)	87 (40.85%)				
Major problem	71 (20.29%)	50 (72.46%)	19 (27.54%)				
Working in the same positions (standing, bending over, sitting, kneeling) for long periods				<0.0001	30	4	34
No problem	39 (11.08%)	12 (30.77%)	27 (69.23%)				
Minimal to moderate	187 (53.13%)	99 (53.8%)	85 (46.2%)				
Major problem	126 (35.8%)	87 (69.6%)	38 (30.4%)				
Bending or twisting your back in an awkward way				<0.0001	30	4	34
No problem	56 (15.91%)	19 (33.93%)	37 (66.07%)				
Minimal to moderate	203 (57.67%)	110 (55%)	90 (45%)				
Major problem	93 (26.42%)	69 (75%)	23 (25%)				
Working near or at your physical limits				<0.0001	30	4	34
No problem	48 (13.64%)	10 (21.74%)	36 (78.26%)				
Minimal to moderate	199 (56.53%)	111 (56.06%)	87 (43.94%)				
Major problem	105 (29.83%)	76 (73.08%)	28 (26.92%)				
Reaching or working away from your body				0.0003	33	4	37
No problem	74 (21.20%)	27 (37%)	46 (63%)				
Minimal to moderate	199 (57.02%)	118 (59.9%)	79 (40.1%)				
Major problem	76 (21.78%)	51 (68%)	24 (32%)				
Continuing to work while injured or hurt				0.0259	31	4	35
No problem	111 (31.62%)	50 (45.87%)	59 (54.13%)				
Minimal to moderate	160 (45.58%)	97 (61%)	62 (39%)				
Major problem	80 (22.79%)	49 (62.03%)	30 (37.97%)				
Lifting or transferring dependent patients				0.0285	35	4	39
No problem	88 (25.36%)	39 (44.3%)	49 (55.7%)				
Minimal to moderate	147 (42.36%)	84 (57.93%)	61 (42.07%)				
Major problem	112 (32.28%)	69 (62.73%)	41 (37.27%)				
Working with confused or agitated patients				0.0497	36	4	40
No problem	92 (26.59%)	47 (52.22%)	43 (47.78%)				
Minimal to moderate	154 (44.51%)	82 (53.25%)	72 (46.75%)				
Major problem	100 (28.90%)	66 (67.35%)	32 (32.65%)				
Carrying, lifting, or moving heavy materials				0.0109	40	4	44
No problem	83 (24.27%)	40 (49.38%)	41 (50.62%)				
Minimal to moderate	167 (48.83%)	89 (53.3%)	78 (46.71%)				
Major problem	92 (26.90%)	63 (70%)	27 (30%)				
Unanticipated sudden movement or fall by patients				0.0746	39	4	43
No problem	134 (39.07%)	64 (48.85%)	67 (51.15%)				
Minimal to moderate	141 (41.11%)	88 (62.41%)	53 (37.59%)				
Major problem	68 (19.83%)	39 (58.21%)	28 (41.79%)				
Assisting patients during gait activities				0.1391	39	4	43
No problem	95 (27.70%)	45 (48.39%)	48 (51.61%)				
Minimal to moderate	170 (49.56%)	98 (57.65%)	72 (42.35%)				
Major problem	78 (22.74%)	48 (63.16%)	28 (36.84%)				
Work scheduling (overtime, irregular shifts, longer length of the workday)				<0.0001	32	4	36
No problem	77 (22%)	27 (36%)	48 (64%)				
Minimal to moderate	182 (52%)	105 (57.69%)	77 (42.31%)				
Major problem	91 (26%)	66 (74.16%)	23 (25.84%)				
Inadequate training on injury prevention				0.0007	36	4	40
No problem	126 (36.42%)	54 (43.55%)	70 (56.45%)				
Minimal to moderate	165 (47.69%)	105 (64.02%)	59 (35.98%)				
Major problem	55 (15.9%)	36 (66.67%)	18 (33.33%)				

Participants with experience of 6-10 years with an average age of 32.63 ±4.53 years are twice as likely as participants with fewer years of experience and an average age of 25.54 ±3.51 years to develop WMSDs (OR: 2.342; 95% CI: 1.062-5.168, p = 0.0350). Table 4 shows the multivariate analysis involving female gender, age, body mass index (BMI), experience, and exercise.

**Table 4:** Predictors of WMSDs among healthcare professionals WMSDs: work-related musculoskeletal disorders; BMI: body mass index; CI: confidence interval; OR: odds ratio

Predictor	Category	Odds ratio	95% CI for OR	P-value
Age	30-50 vs >50	0.872	0.102-7.466	0.9008
	<30 vs >50	0.746	0.075-7.453	0.8031
BMI	Underweight vs obesity	0.826	0.318-2.146	0.6952
	Normal weight vs obesity	1.578	0.704-3.537	0.2684
	Pre-obesity vs obesity	0.743	0.280-1.976	0.5523
Gender	Female vs male	1.173	0.331-4.162	0.8047
Experience	Experience of <1 year	2.415	0.754-7.734	0.1376
	Experience of 1-5 years	1.930	0.797-4.672	0.1448
	Experience of 6-10 years	2.342	1.062-5.168	0.0350
Exercise	Yes vs no	0.864	0.506-1.476	0.5936

The results of the Nordic Musculoskeletal Questionnaire (NMQ), which showed the self-reported symptoms in all the 382 participants, indicated that more than half of the participants (56.66%) had experienced low back pain in the past 12 months, followed by pain in the neck (46.05%), and shoulders (38.81%); for many of them, the WMSDs negatively impacted their performance, leading them to seek help (Table 5).

**Table 5:** Percentages of self-reported musculoskeletal symptoms among all participants (N=382, 100%) MS: musculoskeletal ^*^If the numbers do not add up to the total, it represents missing data, and the shown % figure represents a valid percentage

Body part	^*^Occurrence of MS problems during the last 12 months, n (%)			^*^Normal activity prevented by your problems, n (%)			^*^Seen a physician during the last 12 months for this condition, n (%)			^*^Occurrence of MS problem during the last 7 days, n (%)		
	Yes	No	Total frequency of missing data (n)	Yes	No	Total frequency of missing data (n)	Yes	No	Total frequency of missing data (n)	Yes	No	Total frequency of missing data (n)
Neck	163 (46.05%)	191 (53.95%)	28	62 (18.08%)	281 (81.92%)	39	42 (12.24%)	301 (87.76%)	39	82 (24.26%)	256 (75.74%)	44
Shoulders	137 (38.81%)	216 (61.19%)	29	49 (14.33%)	293 (85.67%)	40	33 (9.62%)	310 (90.38%)	39	54 (16.07%)	282 (83.93%)	46
Upper back	120 (34.09%)	232 (65.91%)	39	42 (12.24%)	301 (87.76%)	39	26 (7.56%)	318 (92.44%)	38	46 (13.69%)	290 (86.31%)	46
Elbows	42 (11.97%)	309 (88.03%)	31	27 (7.89%)	315 (92.11%)	40	14 (4.12%)	326 (95.88%)	42	25 (7.46%)	310 (92.54%)	47
Wrists/hands	93 (26.35%)	260 (73.65%)	29	41 (11.99%)	301 (88.01%)	40	22 (6.47%)	318 (93.53%)	42	44 (13.17%)	290 (86.83%)	48
Lower back	200 (56.66%)	153 (43.34%)	29	99 (28.70%)	246 (71.30%)	37	58 (17.06%)	282 (82.94%)	42	109 (32.54%)	226 (67.46%)	47
Hips/thighs	70 (19.89%)	282 (80.11%)	30	32 (9.33%)	311 (90.67%)	39	17 (5.03%)	321 (94.97%)	44	27 (8.04%)	309 (91.96%)	46
Knees	90 (25.57%)	262 (74.43%)	30	34 (9.94%)	308 (90.06%)	40	24 (7.06%)	316 (92.94%)	42	38 (11.34%)	297 (88.66%)	47
Ankles/feet	98 (27.84%)	254 (72.16%)	30	38 (11.11%)	304 (88.89%)	40	21 (6.25%)	315 (93.75%)	46	40 (11.94%)	295 (88.06%)	47

## Discussion

Work-related musculoskeletal diseases contribute massively to workplace absenteeism as they make employees avoid going to work [16]. These disorders can be more severe among healthcare workers as most of them are engaged in both non-physical and physical work. The objective of this study was to examine the prevalence of work-related MSCs among health practitioners in Al’Qassim, Saudi Arabia, and to identify different risk factors that contribute to the development of WMSDs. We wanted to establish results that can be used by health organizations to assess the problem and execute what is best for healthcare providers.

The total prevalence of WMSDs was found to be 54.7%, which is much higher than in a study done in India, where they reported a prevalence of 26.4% [3]. However, it is a bit lower than what has been found among other studies (79.7%) [13]. This variation can be due to external or internal factors affecting the studies’ results, but the numbers in each study are considered of remarkable significance. The prevalence of MSCs was quite as expected with some variations pertaining to different demographics and work-related risk factors that were considered in other studies.

We found that female practitioners have a higher risk for WMSDs compared to their male counterparts (OR: 1.173; 95% CI: 0.331-4.162), which is similar to a study that was conducted in Turkey and another one from Bangladesh [5,17]. If we look at the work experience and how it correlates with WMSDs, we can appreciate a remarkable variation between different studies; an Indian study concluded that there is no direct link between WMSDs in healthcare providers and their vast years of experience (p = 0.331) [3]. A similar observation was made in another study in Nigeria, which found no relationship between the years of experience and MSCs (p = 0.873) [18]. However, our study concluded otherwise and found that participants with more than six years of experience are at higher risk for developing WMSDs (OR: 2.342; 95% CI: 1.062-5.168; p = 0.0350). This difference between the results can be due to differences in the age of the participants or the greater responsibilities and duties given to senior healthcare providers in our region. Speaking of age, we found that being younger than 30 years makes people less vulnerable to WMSDs (OR: 0.74), and this is similar to what has been found in the Indian study (OR: 0.82); this can be linked with young people's ability to exercise more, as exercise also showed protective results (OR: 0.86). We also found that It is not the number of shifts that can result in WMSDs (p = 0.64), but the duration of the shifts (p = 0.013), and this matches with the findings of a similar study in Jeddah, Saudi Arabia (p = 0.01) [6]. However, this finding differs from what was seen in another study conducted among Indian health practitioners (p = 0.96) [3]. Such differences may be attributed to the difference in the nature of work during shifts in different regions and countries compared to Saudi Arabia.

Among the MSCs that were most frequently reported, low back pain (56.66%) was the predominant complaint, followed by neck pain (46.05%) and pain in the shoulders (38.81%). Pain in the elbow was found to be the least reported complaint (11.97%), and this almost matches with the order of complaints as reported by participants in other studies [3,6]. By observing these numbers and frequencies, we found that low back pain among all is the most frequent complaint that we need to pay special attention to; this MSC has been already studied separately and the results were almost similar to the one we found (70.09%) [19]. This has urged us to propose that healthcare organizations should pay special attention to low back pain. Even though the pain in the lower back, neck, and shoulders was the most frequently reported MSC, few of the participants sought medical help for these complaints during the last 12 months.

Participants reported that most of the job risk factors were causing them trouble and often played a part in their developing WMSDs. Risk factors like performing the same task over and over again (37.96%), working in awkward and cramped positions (20.29%), and working in the same positions (standing, bending over, sitting, kneeling) for long periods (35.8%) were frequently reported. Similar job risk factors were reported by other studies [3,14]. This should trigger the alarm for health education systems around the world to start making healthcare providers pay special attention to what they do at work and maintain healthy physical positioning at work.

This study has some limitations. Similar to some other studies, our study was limited by its cross-sectional design and failure of some participants to answer the questionnaire in full. We recommend conducting a larger, multicentric, prospective study that would include participants from different regions in Saudi Arabia. Such a study will contribute significantly to enhancing the accuracy of the results and understanding WMSDs better. This study and its results should encourage educational programs to develop programs on musculoskeletal disorder prevention strategies as it will reduce the rate of WMSDs and ensure the health and wellbeing of healthcare practitioners.

## Conclusions

As per the findings of this study, we found the highest prevalence of WMSDs in physiotherapists based on the percentage of affected participants from a single specialty. However, nursing was found to be the most affected specialty based on the total number of participants from all specialties. Pain in the lower back, shoulders, and neck were the most frequently reported MSCs. Forceful work, forward bending of the trunk, and neck flexion of more than 20 degrees were found to be the most commonly self-reported ergonomic hazards at workstation relating to the development of WMSDs. We also observed that performing the same task over and over again was the major job risk factor among participants, followed by treating an excessive number of patients in one day.

## Appendices

**Table 6:** Work-related musculoskeletal disorders questionnaire

Questionnaire questions								
Part One (demographic details)								
Personal details:								
What is your age?								
What is your sex?								
What is your height (m)?								
What is your weight (Kg)?								
What is your marital status?								
Occupational and medical history:								
What is your current occupation?								
In which hospital do you work?								
How many years of professional experience do you have?								
How many numbers of shift(s) do you work?								
What is the duration of 1 shift?								
Have you suffered from musculoskeletal pain/discomfort in the past?								
Arthritis?								
Osteoporosis?								
Disk Herniation?								
Low back pain?								
Are you suffering from musculoskeletal pain/discomfort now?								
Lifestyle and habits:								
Do you involve yourself regularly in sports/exercise/yoga?								
Do you exercise regularly?								
Are you a Smoker?								
Are you an Alcoholic?								
Part Two (self-reported ergonomic hazards at workstation):								
Does your job involve neck flexion of more than 20 degrees?								
Does your job involve arm level higher than shoulder?								
Does your job involve repetitive work for more than 4 minutes?								
Does your job involve forceful work?								
Does your job involve the forward bending of the trunk?								
Does your job involve lateral bending or twisting of the trunk?								
Does your job involve prolonged sitting (more than 20 minutes)?								
Does your job involve prolonged standing (more than 20 minutes)?								
Does your job involve lifting, pulling, or pushing?								
Part Three (job risk factor identification) (score of 0 to 10):								
(A score of 0 to 1 indicates that the job factor poses "no problem," a score of 2 to 7 indicates that the job factor poses "minimal to moderate problem," and a score of 8 to 10 indicates that the job factor poses a "major problem")								
Performing the same task over and over?								
Treating an excessive number of patients in 1 day?								
Performing manual orthopedic techniques (joint mobilizations, soft tissue mobilization)?								
Not enough rest breaks or pauses during the workday?								
Working in awkward and cramped positions?								
Working in the same positions (standing, bending over, sitting, kneeling) for long periods?								
Bending or twisting your back in an awkward way?								
Working near or at your physical limits?								
Reaching or working away from your body?								
Continuing to work while injured or hurt?								
Lifting or transferring dependent patients?								
Working with confused or agitated patients?								
Carrying, lifting, or moving heavy materials or equipment (e.g., continuous passive motion)?								
Unanticipated sudden movement or fall by patients?								
Assisting patients during gait activities?								
Work scheduling (overtime, irregular shifts, length of the workday)?								
Inadequate training on injury prevention?								
Part Four (Nordic Musculoskeletal Questionnaire):								
Body part	Have you at any time during the last 12 months had trouble (such as ache, pain, discomfort, numbness) in:		During the last 12 months, have you been prevented from carrying out normal activities (e.g., job, housework, hobbies) because of this trouble in:		During the last 12 months, have you seen a physician for this condition:		During the last 7 days, have you had trouble in:	
	Yes	No	Yes	No	Yes	No	Yes	No
Neck								
Shoulders								
Upper back								
Elbows								
Wrists/hands								
Lower back								
Hips/thighs								
Knees								
Ankles/feet								

**Table 7:** Informed consent form given to participants

Informed Consent for Research: Work-related Musculoskeletal Disorders Among Medical Practitioners at Hospitals of Al’Qassim Region, Saudi Arabia
Voluntary participation: participation in this study is voluntary. You will suffer no penalty nor loss of any benefits to which you are otherwise entitled should you decide not to participate. Withdrawal from this research study will not affect you in any way
Confidentiality: Your identity and medical record, as a participant in this research study, will remain confidential with respect to any publications of the results of this study
Authorization of voluntary participant who is not expected to obtain any direct benefit
A: I acknowledge that I have (read/or had explained to me) in a language I understand, the attached research participant Information sheet and that Dr………………..has explained to me the nature and purpose of this study. I have had the opportunity to ask any questions I had with respect to this study and all questions I asked were answered to my satisfaction
B: I understand the purpose of this study and I voluntarily accepted it after sufficient explanation
C: I understand that I am free to withdraw this authorization and discontinue participation in this study at any time. The consequences and risks, if any, of such withdrawal during the course of the study have been explained to me
D: I confirm that I have (read/or had read to me), the foregoing authorization and that all blanks or statements requiring completion were properly completed before I signed
I confirm that I have accurately (translated and/or read) the information to the subject: Signature: Participant name: Hospital ID#:
